# Observations on the Cure of the Gonorrhœa

**Published:** 1781-10

**Authors:** 


IV. *Obſervations on the cure of the Gonorrhœa. By* Samuel Foart Simmons, M. D. *Member of the Royal College of Phyſicians, London; and* F. R. S. 8vo. Murray, *London,* 1780. 70 pages, 1s. 6d.

ALTHOUGH the venereal diſeaſe has prevailed in Europe for more than two hundred years, we find men of the greateſt eminence in the profeſſion ſtill differing in their opinions concerning its nature, and in their methods of treating it. In this ſtate of doubt, a publication, like the preſent, written with candour, and founded on attentive obſervation, cannot but prove highly acceptable to the medical reader. The author profeſſes to have carefully avoided all ſpeculative reaſonings, and to have confined himſelf wholly to ſuch practical cautions and facts as are derived from his own experience.

It has lately been ſuggeſted, that the gonorrhœa and the lues venerea are different affections, originating from two diſtinct ſpecies of virus. Dr. Simmons contends, that this opinion is ill founded, and obſerves, that the matter of a chancre introduced into the urethra will generate a gonorrhœa, and that the diſcharge of a gonorrhœa will produce a chancre, bubo, and lues. Two caſes are related in which ſymptoms of a confirmed lues venerea were brought on by the imprudent ſuppreſſion of the diſcharge in a gonorrhœa.

It has been diſputed, whether ulceration is ever produced in the urethra by a gonorrhœa, and of courſe whether an abſorption of the matter can take place. It might be ſuppoſed that diſſection would at once clear up this matter, but this is ſo far from being the caſe, that ſome of

ſome of the moſt celebrated anatomiſts are found to entertain different opinions on the ſubject. Our author has opened the urethras of ſeveral perſons, who had a gonorrhœa at the time of their death, but the appearances were not ſufficiently ſatisfactory to enable him to decide with certainty on the ſubject. On the other hand he has met with ſeveral inſtances in his diſſections, and has ſeen others in the collections of different anatomiſts, of urethras that afforded evident marks of cicatrices from ulcers formerly exiſting in that canal. And when we conſider that the diſcharge in a gonorrhœa is ſometimes tinged with blood, and that when this happens, a little blood veſſel is no doubt ruptured; he thinks we can have no reaſon to doubt that an ulceration may, and ſometimes does happen in theſe caſes. It is certain—he ſays—that whereever there is conſiderable inflammation there will be danger of ulceration. He thinks that ſlight ulcerations of the urethra may often occur, and be afterwards perfectly obliterated in a ſimilar manner to what happens in the papillæ of the tongue, the tonſils, &c. Such an obliteration, he obſerves, will the more readily take place in a part like the urethra, defended with mucus

 and and not expofed to the air, which is known to have no little effect in hardening a cicatrix.

After difcuffing this point in a very fatisfactory manner, Dr. Simmons proceeds to defcribe the fymptoms and progrefs of the difeafe with great accuracy, and then points out the means of cure. The principal indications, he obferves, are to fubdue the inflammation and remove the virus that occafions it.

There are practitioners, who, fuppofing that the body poffeffes powers to expel the virus, and that the difeafe has a certain period to run, through its feveral ftages of progrefs, acme, and decline, are for leaving the cure to nature, or at leaft content themfelves with affifting her by an antiphlogiftic regimen, gentle evacuations, and the like. That in many cafes the diforder admits of a natural cure, Dr. Simmons thinks there can be no doubt; the increafed fecretion of mucus carrying off the virus fafter than it is formed, till at length the infection is wholly removed. But he is of opinion, that in every cafe by the application of fuitable remedies, to the inflamed part, we may fhorten the duration of the complaint, and abridge the fufferings of the patient with the fame certainty and fafety, as we are enabled to remove the effects of an ophthalophthalmia, or any other local inflammation, by proper topical applications.

Dr. Simmons next ſpeaks of the general remedies employed in theſe caſes, ſuch as occaſional blood letting, a cooling diet, the liberal uſe of diluting liquors, and mild purges. Of the firſt of theſe, venæſection, he remarks, that it is ſeldom requiſite. He has ſeen it of uſe in ſtrong, plethoric patients, when the chordee has been frequent and painful; but he has hardly ever found it neceſſary to repeat the operation. He obſerves, that the inflammation in theſe caſes is kept up by the local ſtimulus of the virus and the urine, ſo that all that we can expect from venæſection is to moderate the pain and the frequency of erection. In delicate perſons he has ſeen it do harm by increaſing irritability and rendering the patient more ſuſceptible of the ſtimulus. In treating of diet Dr. Simmons points out the advantages of an antiphlogiſtic regimen, and recommends a free uſe of mucilaginous liquors, but objects very properly to the uſe of nitre or cream of tartar on account of their diuretic property. Our view, he obſerves, being not to promote a preternatural flow of urine (as the virus, being inſoluble in water, cannot be waſhed away by ſuch means) but to render render that which is secreted as mild and as little stimulating as possible.

He next treats of purgatives, which when administered with prudence he considers as of great use, but it is well known —he observes— that the abuse of this class of remedies in the gonorrhœa has been productive of numerous evils. He advises us to employ gentle laxatives, such as Rochelle salt, manna, soluble tartar, and the like, in a dose sufficient to procure only two or three stools, and repeated only every two or three days. The daily use of the purgative electaries, that are still given by some practitioners, serve only to keep up a continual irritation on the bladder, and of course to prolong the inflammation. Formerly it was a pretty general practice to give a large dose of calomel at bed-time, three or four times a week, and to put it off the next morning with a strong dose of the *pilulæ cocciæ*, or some other drastic purge. Our author observes, that as the constant effect of a violent purge is to promote absorption from every cavity, the venereal virus was by this means frequently carried into the system, and produced a confirmed lues; or, if the patient escaped this evil, he at least found himself troubled with an obstinate gleet, and, perhaps, his his conftitution materially injured. He adds alfo, that violent purging often occafions ftrangury, hernia humoralis, and other troublefome fymptoms.

The topical remedies that are ufed in gonorrhœa confift chiefly of various forts of injections, the ingredients of which are extremely various; but Dr. Simmons is of opinion, that their modes of operation may in general be referred to their mucilaginous and fedative, or to their detergent, ftimulating, and aftringent qualities. He obferves, that in the hands of fkilful practitioners great advantages may doubtlefs be derived from the ufe of thefe remedies; but on the other hand, that the improper and unfeafonable adminiftration of them may prove a fource of irreparable mifchief to the patient.

Dr. Simmons points out the advantages and difadvantages of the feveral kinds of injections, and gives us a variety of practical cautions concerning the ufe of each, for which we muft refer our readers to the work itfelf. Speaking of mercurial injections, he obferves, that they have all of them more or lefs of aftringency, and it is folely to this property that he afcribes their effects, as he fuppofes that the idea of their correcting the venereal virus was originally introintroduced, and has been continued upon miſtaken principles. He obſerves, that calomel, mixed with the mucus diſcharged in a gonorrhœa, has no more power in deſtroying the infectious properties of that mucus, than ceruſſe or any other preparation would have; that a diluted ſolution of ſublimate, injected into the urethra, will, like a ſolution of verdegris, or blue vitriol, or any other ſtyptic, conſtringe the mouths of the lacunæ; but that this is all it will do, as it will never leſſen the infectious property of the virus. He contends, that mercury has no power over the venereal virus, until it has been introduced into the body, and undergone certain changes, with which we are and probably ſhall ever remain unacquainted.

In caſes of gonorrhœa whenever our author has adminiſtered mercury internally, it has ſeldom been with a view to expedite the cure, but merely to obviate the danger of abſorption. When the infection has been apparently ſlight, and the inflammation and other ſymptoms trifling, he has ventured to proceed without the aſſiſtance of mercury. On the other hand, whenever the diſcharge was conſiderable, the inflammation violent, or the ſeat of the diſeaſe high up in the urethra, he has conſtantly judged it adadvifeable to give fmall dofes of calomel or of the mercurial pill of the Edinburgh Difpenfatory. When there is no chancre or bubo, no appearance, in fhort, that the infection is likely to be carried into the fyftem, he thinks it would be imprudent to adminifter corrofive fublimate, or any other of the more acrid preparations of mercury.

After having treated very fully on the general remedies employed in the cure of the gonorrhœa, Dr. Simmons proceeds to fpeak of hernia humoralis, chordee, bubo, phymofis, and paraphymofis, chancres, ftrictures of the urethra, and gleets.

He confiders the hernia humoralis as being merely the effect of irritation and of increafed inflammation. He obferves that in the greater number of cafes the inflammation is confined to the vas deferens and epididymis, the tefticle itfelf being feldom affected. So little connection has it with the flow from the urethra, that we are told it fometimes comes on during the continuance of the difcharge. Sufpenfion of the fcrotum, an horizontal pofture, venæfection, warm bathing, and cold applications, fuch as cloths dipped in vinegar, to the part, are the general means of cure recommended by our author in cafes of this fort.

In chordee he has often experienced the good effects of leeches applied near to the feat of inflammation, but he obferves, that the beft method is to obviate the complaint by confining the penis in fuch a manner as to prevent erection. He fpeaks of a fpafmodic chordee which fometimes remains after all the other fymptoms of gonorrhœa have difappeared, and goes off and returns at times for the fpace of feveral months. In general he has found this troublefome complaint give way to a liberal ufe of the Peruvian bark fooner than to any other remedy.

In treating of buboes Dr. Simmons obferves, that when a tumour of this fort has once begun to form, it is the opinion of the generality of practitioners, that its fuppuration ought to be encouraged, left by difperfing it the matter be carried into the fyftem, and fo produce a confirmed lues. He offers feveral obfervations on this fubject, which feem to prove beyond a doubt that fuch an event is much more likely to happen by promoting, than by preventing the fuppuration of the tumour. As the moft effectual means of difperfing it he recommends cold applications to the part; but the moft powerful remeremedies, he observes, in such cases, are vomits. As a further proof that mercury acts only by its stimulus, we are told, that mercurial unction rubbed on the inside of the thigh will frequently increase the inflammation, and of course hasten suppuration, and promote the very end it is intended to prevent.

In opening a bubo our author prefers a caustic to the knife, but he has generally found that when the patient has been of a good habit of body, and the tumour has maturated quickly, it has, when suffered to break of itself, healed sooner than it usually does when opened either by caustic or the knife.

In the treatment of a chancre, if it is small, and without any considerable inflammation, he advises the touching it repeatedly with the lunar caustic, so that the sore shall throw off sloughs. By this process, he observes, we *destroy* the venereal virus, instead of *repelling* it, which might be the case if the caustic were not of sufficient activity to produce a slough, for in that case it would only stimulate, and of course serve to throw the virus into the habit. He asserts, that the blue ointment when applied to a chancre has no other property than any other unctuous substance would have, except what it owes to

 its its ſtimulus; and that red precipitate in a ſimilar caſe will act only as blue vitriol or any other eſcharotic would do. Although in the greater number of caſes chancres are at firſt only local affections, Dr. Simmons thinks it prudent to guard againſt the poſſibility of the virus infecting the habit by giving mercurials internally.

Our author ſuppoſes the exiſtence of caruncles in the urethra to be a very rare occurrence. He conſiders a ſtricture or contraction of ſome part of the canal as being the moſt general cauſe of the obſtruction. He thinks, that the cure of this ſtricture by bougies depends ſolely on their producing a gradual diſtenſion of the membrane. In the work itſelf the reader will find a great number of uſeful remarks on the management of bougies. Dr. Simmons has ſometimes met with caſes of ſtricture that were only temporary, and ſeemingly owing to a ſpaſmodic affection from increaſed irritability. This ſpaſmodic ſtricture, like the ſpaſmodic chordee, generally gave way to the bark.

Dr. Simmons concludes his work with ſome remarks on gleets, in the cure of which, when there is no reaſon to ſuſpect any venereal taint, we are adviſed to employ aſtringent injections. It will be neceſſary, we are told, to attend at the

