# Errata

**Published:** 1781-12

**Authors:** 


					?p. 238, 1. li, for ' put it off* rW
' purge it off.'
I N D E ^

				

